# K-edge Subtraction Computed Tomography with a Compact Synchrotron X-ray Source

**DOI:** 10.1038/s41598-019-49899-z

**Published:** 2019-09-16

**Authors:** Stephanie Kulpe, Martin Dierolf, Benedikt Günther, Madleen Busse, Klaus Achterhold, Bernhard Gleich, Julia Herzen, Ernst Rummeny, Franz Pfeiffer, Daniela Pfeiffer

**Affiliations:** 1 Chair of Biomedical Physics, Department of Physics, Technical University of Munich, James-Franck-Straße 1, 85748 Garching, Germany; 2 Munich School of BioEngineering, Technical University of Munich, Boltzmannstraße 11, 85748 Garching, Germany; 3 Department of Diagnostic and Interventional Radiology, School of Medicine & Klinikum rechts der Isar, Technical University of Munich, Ismaninger Straße 22, 81675 München, Germany

**Keywords:** Radiography, Preclinical research, X-rays, Imaging techniques

## Abstract

In clinical diagnosis, X-ray computed tomography (CT) is one of the most important imaging techniques. Yet, this method lacks the ability to differentiate similarly absorbing substances like commonly used iodine contrast agent and calcium which is typically seen in calcifications, kidney stones and bones. K-edge subtraction (KES) imaging can help distinguish these materials by subtracting two CT scans recorded at different X-ray energies. So far, this method mostly relies on monochromatic X-rays produced at large synchrotron facilities. Here, we present the first proof-of-principle experiment of a filter-based KES CT method performed at a compact synchrotron X-ray source based on inverse-Compton scattering, the Munich Compact Light Source (MuCLS). It is shown that iodine contrast agent and calcium can be clearly separated to provide CT volumes only showing one of the two materials. These results demonstrate that KES CT at a compact synchrotron source can become an important tool in pre-clinical research.

## Introduction

Computed tomography (CT) is one of the most important imaging techniques in clinical diagnostics. Since its development in 1973 by Hounsfield^1^, the number of CT procedures performed, e.g., in the United States alone has reached approximately 74 million in 2017^2^. Technical advances in the past decades have increased the clinical impact of CT and significantly enhanced its widespread use in diagnostics^3^. Among these developments is contrast enhanced CT.

**Figure 1:**
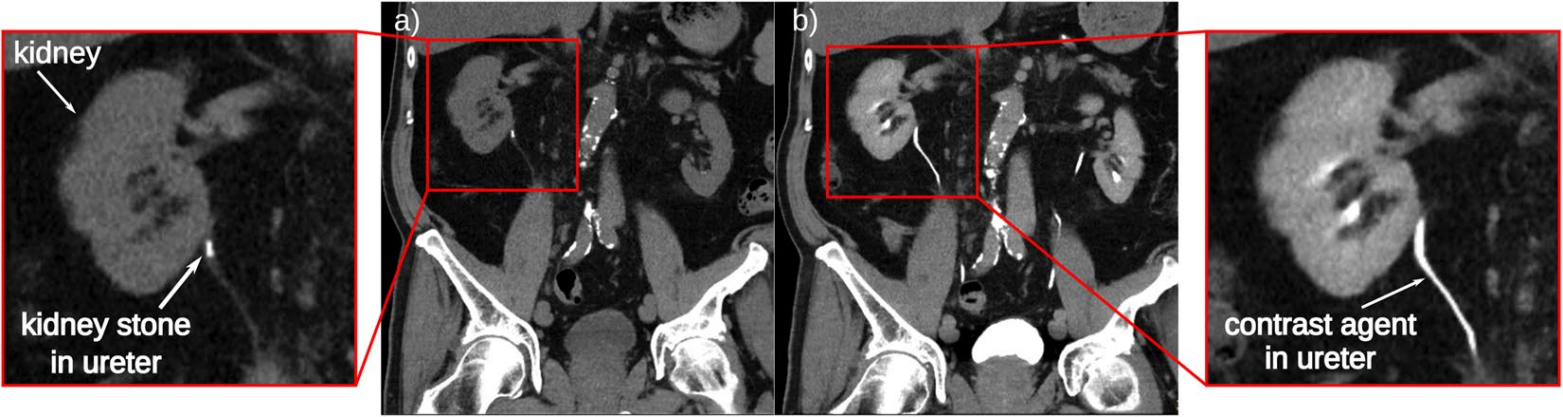
Clinical X-ray CT images before (**a**) and after (**b**) administration of iodine contrast agent. In the CT image in (**a**) the kidney stone is clearly visible in the proximal part of the right ureter. However, after the injection of contrast agent (**b**) the kidney stone in the ureter cannot be distinguished from the iodine contrast agent and would be missed.

This is used e.g. in renal imaging, where iodine contrast agent is commonly applied for examinations of the renal parenchyma (e.g. for detection and characterization of tumors), to illustrate the arterial blood supply of the kidneys (e.g. for detection of artery stenosis or bleeding) and to visualize the urinary tract using delayed phase images, when the contrast agent passed the kidney and is visible within the ureter (e.g. to rule out ureteral obstruction). Fig. 1(a) shows CTs of a patient with a kidney stone in the ureter (indicated by the white arrow), which could block urine flow. To decide on further treatment, iodine contrast agent is injected into the blood stream to contrast the urine flow through the ureter after the contrast agent has been filtered from the blood. Yet, the contrast agent in the ureter gives a similar absorption signal as the kidney stone so that the discrimination of the two can only take place by taking two CT scans, a non-contrast scan before the injection of the contrast agent and one with contrast agent in the excretory phase. These have to be taken with a sufficient time span between them to ensure having contrast agent in the ureter in the contrasted scan.

In conventional X-ray imaging, the image contrast arises from variations in absorption of different materials. Thereby, the absorption is dependent on the elemental composition and density of the material, leading to a strong contrast between bone and tissue structures. Yet, the elemental composition of soft tissues is rather similar and the attenuation contrast is weak. In subtraction X-ray imaging, tissue structures or organs are visualized using a contrast medium that changes the attenuation between the contrasted structure and the surrounding tissue^4^. In K-edge subtraction (KES) imaging, first proposed by B. Jacobson in 1953^5^, two X-ray images are taken at different mean energies slightly below and a bit above the K-edge of the photoelectric absorption of the contrast medium. Their subtraction generates an image only displaying the contrasted structure while other anatomical structures or bones are eliminated as their attenuation stays basically constant. This can solve issues where the attenuation of a tissue was enhanced such by a contrast agent that it becomes indistinguishable from another structure. Commonly, this is the case with iodine-based contrast agent and calcium in calcifications or kidney stones (Fig. 1b). Although KES imaging at a polychromatic laboratory source has been demonstrated recently using a Ross filter arrangement^6^ or a multi-bin photon counting detector^7,8^, there are disadvantages such as the absorption of a large amount of the X-ray flux in the filter pairs leading to long acquisition times. Also the development of 2D pixelated spectroscopic detectors allow KES imaging at a laboratory X-ray source^9,10^. However, in some of these applications the sample is irradiated with a much broader beam than needed for the selected energy bins causing unnecessary radiation dose. In the past, KES imaging has usually been performed at synchrotrons.

Synchrotrons providing highly brilliant monochromatic X-rays commonly used for KES imaging offer several advantages for CT compared to conventional broad X-ray spectra. Besides generating a significantly higher photon flux, beam hardening artifacts are absent^11,12^. After some major improvements in image quantification, elemental sensitivity and soft tissue contrast, Dilmanian *et al*.^13^ performed the first temporal K-edge subtraction CT. Since then, KES imaging has been an established technique at synchrotron facilities. Most of the K-edge imaging methods at synchrotrons use a beam that is significantly wider in the horizontal direction so that the sample has to be scanned in vertical direction, allowing for µCT applications^14^. Others use X-ray optics to expand the beam vertically^4^, which enables investigation of larger samples. In several feasibility studies, it has been shown that monochromatic KES CT provides improved image quality and quantitative accuracy compared to conventional polychromatic CT^15–17^. Additionally, the use of special monochromators allows for the simultaneous acquisition of both CT scans below and above the K-edge, providing an advantage for the imaging of living specimens^18,19^.

Although KES CT has been successfully performed at synchrotrons in the past, the integration of synchrotrons into a clinical setting is difficult. Not only are synchrotrons expensive both in installation and maintenance, also their size further limits their usage for clinical applications. Additionally, the typical beam size provided by a synchrotron is far below the beam size needed for clinical diagnostics. Over the past decades, Compact Synchrotron Sources have been developed as an alternative means to serve the need for high monochromatic flux X-ray beams and may have the potential to make KES imaging available in a clinical setting in the future^16^. Currently many projects to build inverse Compton sources are ongoing, such as e.g. the cERL based laser-Compton X-ray source at KEK^20^, STAR^21^, the ASU Compact XFEL^22^ and ThomX^23^. One major advantage of inverse Compton sources is that they provide high brilliance X-ray beams with reduced financial and spatial requirements^24,25^. Also, at the Munich Compact Light Source (MuCLS), a storage ring-based inverse Compton source is used to produce an X-ray beam with the aforementioned characteristics. It has already been shown that a quasi-monochromatic CT performed at an inverse Compton source provides improved image quality in comparison to CTs taken at a conventional rotating anode source^26^.

To enable KES imaging at the MuCLS the energy of the source is changed by the insertion of an iodine filter. The X-ray spectrum is tuned such that the mean energy of the unfiltered spectrum lies directly above the iodine K-edge. When filtering the spectrum, the part of the spectrum above the K-edge of iodine is absorbed by the filter, thus shifting the mean energy of the remaining spectrum below the K-edge. With this set-up, it has been shown that KES imaging in projections is beneficial for the contrast-to-noise ratio of small blood vessels overlaid by bone structures^27^. To further explore the advantages of this new X-ray source, the imaging setup has been further developed to enable KES CT.

Here, we present a proof-of-principle of KES CT of a porcine kidney at the MuCLS. For this, two CT scans were performed, one with the unfiltered spectrum of the MuCLS and a second with the iodine filtered spectrum. We demonstrate the differentiation of iodine-based contrast agent from a calcium-based kidney stone. It is shown that KES CT solves the clinically faced problem of indistinguishability of iodine and calcium.

## Results

**Figure 2:**
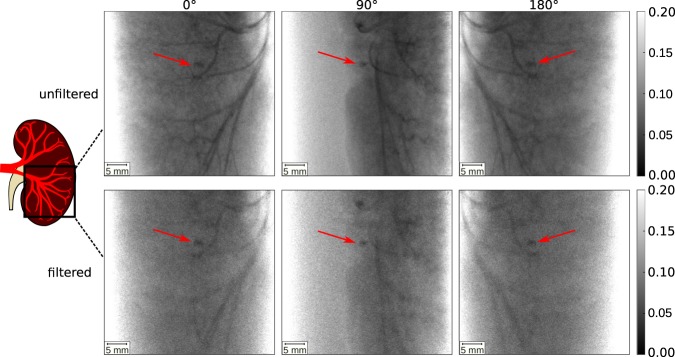
MuCLS X-ray projection images filtered and unfiltered CTs at projection angles 0°, 90° and 180°. The kidney stone is indicated by a red arrow. In the unfiltered projection images, the iodine filled blood vessels are clearly visible together with the kidney stone. In the filtered projection images, the X-ray attenuation of the iodine contrast agent is reduced, yet still the differentiation of iodine and the kidney stone is difficult. The gray scales of the projection images show the relative transmission of the X-ray beam.

In Fig. 2, X-ray projection images of both the unfiltered and filtered CT scan of a porcine kidney together with a kidney stone are presented. The kidney can be seen from the front (0°), side (90°) and back view (180°). In the unfiltered images, the renal arteries filled with iodine contrast medium are clearly visible. The kidney stone, indicated by the red arrow, absorbs similarly to the iodine in all projections which makes the differentiation of the two difficult. The absorption of the kidney stone was calculated exemplary in the projection at 0° to 0.956 ± 0.006, the absorption of the iodine is 0.953 ± 0.004. With a single CT scan, the two materials cannot be separated. Therefore, both, clinical imaging and the KES CT method, require a second scan. In contrast to clinical imaging, where the second scan is time-delayed in order to acquire scans with contrast agent and without it, the X-ray spectrum is filtered in our approach to change the mean energy for the second scan. In the filtered projection images, the X-ray absorption of the iodine contrast agent is reduced to 0.937 ± 0.007 due to the absence of the high energy photons above the K-edge in the spectrum. The absorption of the kidney stone in the filtered projection image is 0.9504 ± 0.007 and barely changes in comparison to the unfiltered images.

**Figure 3:**
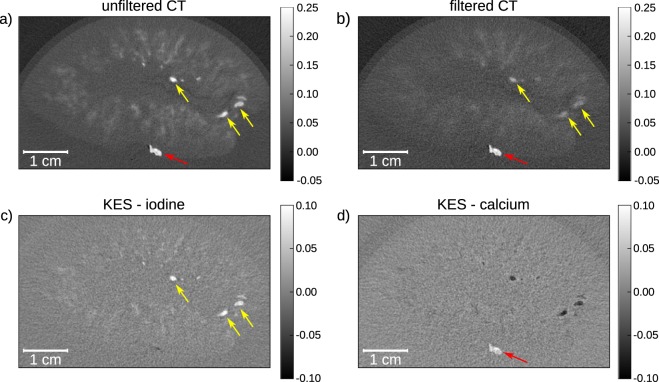
Reconstructed CT slices of porcine kidney with kidney stone (indicated by the red arrow) in transverse slice orientation (slice thickness: 70 µm). (**a**) Unfiltered CT slice, where both the blood vessels (indicated by the yellow arrows) and the kidney stone are visible; (**b**) iodine filtered CT slice, where the attenuation of the iodine in the blood vessels is reduced, yet it is not possible to distinguish the two materials; (**c**) in the KES image only structures containing iodine contrast agent stay visible, the kidney stone is eliminated from the image; (**d**) when performing inverse KES, the iodine is inverted so that the kidney stone can be clearly identified. The gray scales of the unfiltered and filtered CT slices show the absorption values in 1/mm whilst the KES slices show the differences in absorption.

In Fig. 3, the reconstructed slices through the kidney and the calculated KES iodine and inverse KES calcium slices are presented. In both the unfiltered CT (Fig. 3a) and the filtered CT slice (Fig. 3b) the iodine filled blood vessels inside the kidney are visible. Additionally, the diffusion of the iodine into the tissue can be seen as slightly contrasted areas. The attenuation coefficient µ of the calcium-based kidney stone (0.2105 ± 0.0151 1/mm), which is positioned on the surface of the kidney, is very similar to the attenuation of the contrast agent (0.2184 ± 0.0100 1/mm). As the kidney stone would be located inside the renal pelvis or ureter in a patient setting, the iodine contrast agent and the kidney stone are expected to be in the same area, while similar CT values would make it impossible to separate both materials. After K-edge subtraction of the two slices (Fig. 3c), every material but iodine is eliminated from the resulting image so that the blood vessels can be clearly identified. In the inverse KES (as described in the Methods section), the kidney stone becomes visible whilst the iodine filled blood vessels are inverted (Fig. 3d). The use of the KES subtraction method for CT allows to separate iodine contrast agents and calcifications. This is especially helpful in clinical diagnostics when looking for kidney stones in contrast enhanced CT.

**Figure 4:**
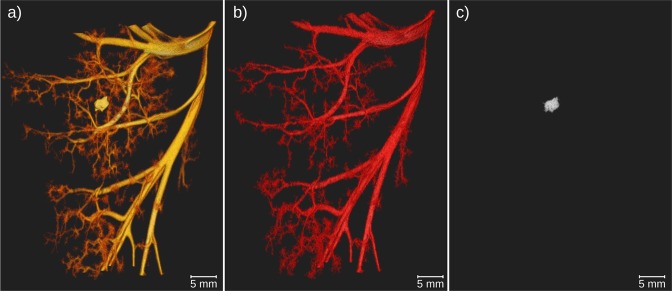
3D visualization of segmented CT data to visualize solely the hyperdense structures. (**a**) The blood vessels and kidney stone were segmented from the unfiltered CT scan. The differentiation of the two materials is not possible; (**b**) blood vessels were segmented from the iodine KES data, in which the kidney stone is not visible; (**c**) by performing inverse KES the calcium within the kidney stone becomes visible again whilst the iodine-filled blood vessels are subtracted from the image.

The CT data can be segmented such, that similar absorption values can be separated from the surrounding structures. This was done for the unfiltered CT scan and the calculated KES iodine and inverse KES calcium scans and is presented in Fig. 4(a–c), respectively. In the unfiltered CT scan in Fig. 4(a), the attenuation values of iodine and calcium are very similar, so that they cannot be separated by a simple histogram-based segmentation. In the KES iodine image in Fig. 4(b), the blood vessels can be clearly identified. In Fig. 4(c), solely the kidney stone is visible. Thus, also in the segmentation of the CT data, the KES method helps to distinguish blood vessels and calcifications such as kidney stones.

## Discussion

The results of this proof-of-principle experiment demonstrate that KES CT is feasible with the quasi-monochromatic spectrum at the MuCLS using a filter-based K-edge subtraction method. Taking two CT scans at different mean energies, it was possible to calculate images separating iodine contrast agent and calcium, a commonly faced issue in clinical imaging. However, currently, the scan time of the CT obtained at MuCLS is not compatible with the needs in a clinical routine. A full body scan can be performed in down to 5 s at a clinical CT device^3^. At the MuCLS, the scan was performed at a multi-purpose research setup not optimized for one specific application. Therefore, the scan time is currently around 10 min but could be further reduced by optimizing the detector communication, using a larger detector pixel size and allowing a continuous movement of the sample stage. This would provide the basis for further experiments such as *in-vivo* small animal KES CT. With additional development of the imaging setup and the image processing algorithms KES imaging can provide further advantages for CT imaging. Monochromatic imaging allows elemental decomposition at a higher accuracy than state-of-the-art clinical imaging^13^. In addition, the absolute concentration of the contrast agent^28–30^ can be determined in KES CT as well as the electron density of the material after performing a dual-energy approach^31–33^. In most KES CT synchrotron studies the clinical applicability was limited by the dose applied to the specimen, with dose levels between 7 mGy^33^ and 9 Gy (skin entry dose)^19^. Yet, KES imaging at synchrotrons has been demonstrated with clinically acceptable dose levels in transvenous coronary angiography^29^ and in *in-vivo* KES CT^16^. The effective dose in this experiment of 2.67 mSv was far below the dose applied in clinical dual energy CT, where dose levels of 5–12 mGy (CTDI) per scan are achieved^34–37^. Also, the effective dose level in this experiment was lower than effective doses usually applied in renal angiographic CT being around 5 mSv^38^. However, here, only a single organ was imaged.

The clinical applicability of KES CT at the iodine K-edge is limited due to the strong attenuation of X-rays in the human body at ~33.7 keV X-ray energy. This proof-of-principle study was performed with an iodine contrast agent as it is the standard contrast agent in clinical CT, with the K-edge of iodine at 33.17 keV. Especially for patients with severe iodine allergies, the shift to other contrast agents as e.g. gadolinium would be an option. In the past, there have been promising results showing the feasibility of using gadolinium-based contrast agents for angiography^39–41^. With the K-edge of gadolinium located at 50.2 keV, this would require higher X-ray energies for KES CT. In general, there is no limit on the achievable X-ray energy of inverse-Compton sources. When using larger electron storage rings and higher electron energies, higher X-ray energies than 50 keV can be achieved. Several inverse Compton sources with these higher X-ray energies are in development, such as STAR^21^ and ThomX^23^. Alternatively, by increasing the energy of the laser photons used in the inverse Compton process, higher X-ray energies become also accessible at lower electron energies, i.e. with a machine size similar to the MuCLS. This ongoing development of compact synchrotron sources will extend the applications of these X-ray sources and allow methods that are currently used at synchrotrons to be transferred to a laboratory environment^42^. This will allow KES imaging to become an important tool for biomedical research on potential clinical applications^4^ and provide the basis for dose compatible KES CT imaging in the future.

In conclusion, it has been shown that KES CT is feasible at a compact synchrotron X-ray source which is going to provide benefits for contrast enhanced 3D imaging in a pre-clinical setting. KES CT allows for a discrimination of iodine and calcium, which will be of special interest in various clinical situations like kidney stones, atherosclerosis and bone imaging.

## Materials and Methods

### Working principle of the Munich Compact Light Source

The Munich Compact Light Source consists of a compact synchrotron source (developed and manufactured by Lyncean Technologies Inc., USA), and an in-house developed dedicated imaging beamline with two end-stations. The source is based on inverse Compton scattering, i.e. it produces X-rays by collision of infrared laser photons with relativistic electrons, thereby providing a tunable, quasi-monochromatic X-ray beam^24,26^.

To ensure a continuously high X-ray flux, the electrons orbit in a storage ring while the laser pulse is stored in a high finesse optical resonator. Their revolution frequencies (65 MHz) are matched so that electrons and laser photons collide upon each revolution at the interaction point.

In the approximation of head-on collision of laser photons with electrons and backscattering of the X-ray photons, the X-ray energy is given by E_X_ ≈ 4γ^2^E_L_, with γ = E_e_/mc^2^ being the ratio of electron energy to electron rest energy and E_L_ the laser photon energy^43^. The X-ray energy is tunable between 15 and 35 keV by changing the electron energy. The X-ray beam is quasi-monochromatic with a bandwidth below 4.5% full width half maximum and partially coherent. The X-ray flux of the source is up to 3.5 × 10^10^ photons per second^44^ (at 35 keV) with a source size of about 45 × 45 μm^2^. The X-rays are emitted into an opening angle of 4 mrad, so that the beam has an elliptic extent of 62 × 74 mm² at the detector position.

**Figure 5:**
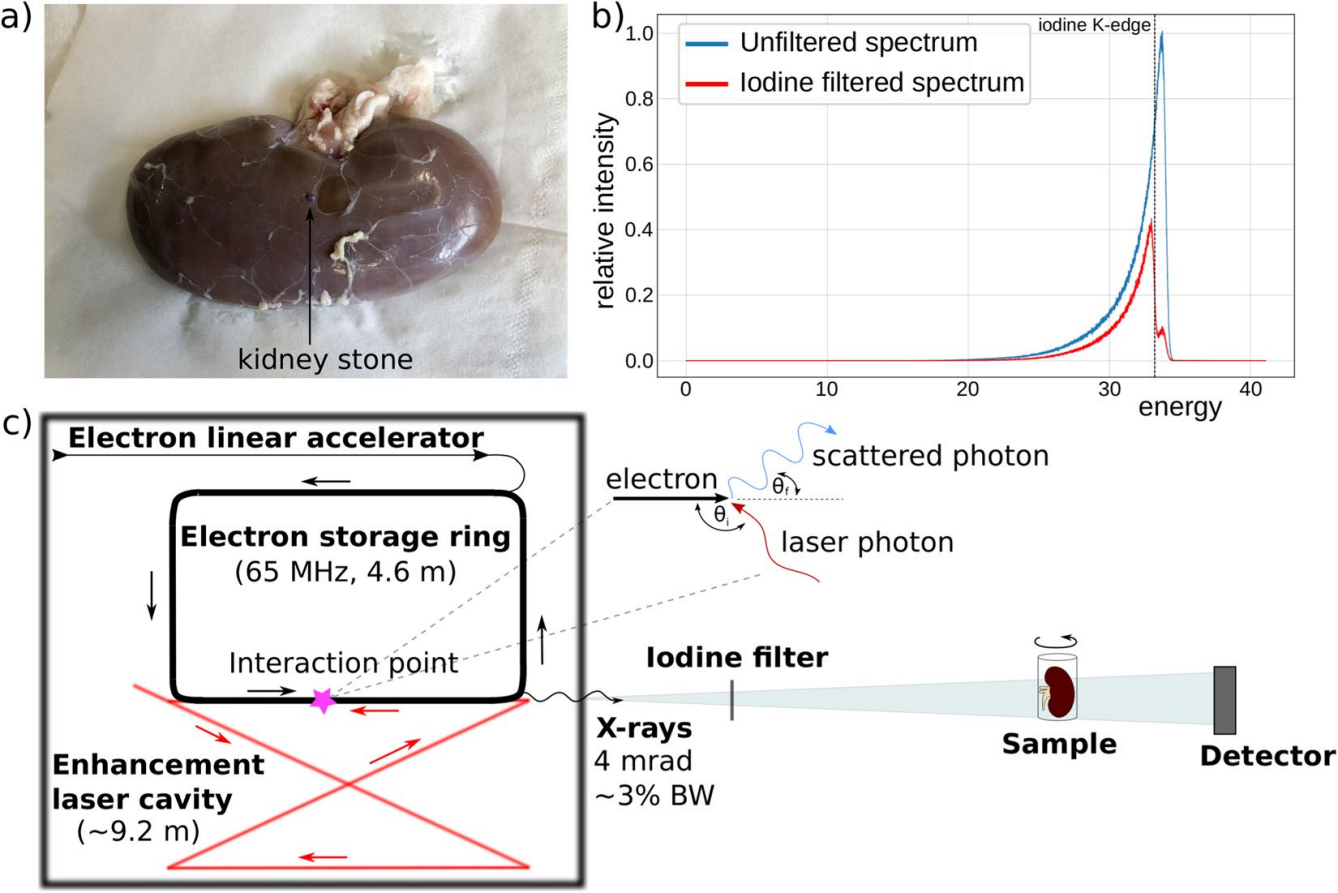
(**a**) Photography of the porcine kidney and the kidney stone used in experiment with a human kidney stone placed on the outside of the kidney, (**b**) Plot showing the unfiltered spectrum of the MuCLS together with the iodine filtered one and the iodine K-edge, (**c**) Schematic of MuCLS and experimental set up: The source is placed in a radiation safe cave from which the X-ray beams exit into a evacuated pipe. The iodine filter is placed into the beam if needed at a distance of 3.5 m from the source. The sample is set at a source-to-sample distance of about 15.3 m and the detector is located 16.4 m from the source point.

The experiments were performed at the far end-station of TUM’s imaging beamline. The sample (see Fig. 5a) was placed at a source-to-sample distance of about 15.3 m and the detector was located 16.4 m from the source point. The iodine filter was placed at a distance of 3.5 m from the source in the near end-station of the imaging beamline. The source together with the experimental setup is shown in (Fig. 5c).

### K-edge subtraction computed tomography

While the X-ray energy at the MuCLS can be adjusted by changing the electron beam energy, this process requires a concerted change of a multitude of machine parameters. A quick oscillation of the electron beam energy, therefore, requires operating the machine in a non-equilibrium state, which makes it non-trivial. Therefore, the energy change of the spectrum was performed with an iodine filter. Furthermore, this iodine filter approach in principle allows for sub-second switching of the energy spectrum, while a switch through the electron beam energy – due to the many parameters that have to be changed – will probably be feasible in a second.

The X-ray spectra used for the experiments are shown in Fig. 5(b). The MuCLS was tuned to a peak energy of 33.69 keV such that the mean energy of the unfiltered spectrum was 33.18 keV, just above the K-edge of iodine. By filtering the spectrum, the high energy part of the spectrum above the K-edge was absorbed so that only 3% of the intensity of this high energy part of the spectrum remained. The filtered spectrum then had a mean energy of 32.59 keV. The energy separation of the two scans is therefore 0.59 keV. Since Sarnelli *et al*.^45^ showed that a smaller energy separation is beneficial for the contrast in the KES image, this energy separation is considered ideal for the experiment. For the experiments, a solid iodine filter was used which was made of an iodine-based contrast agent (Ultravist 370, Bayer Schering Pharma AG, Germany) embedded into a PVP polymer-matrix. The effective iodine thickness of the filter is ~290 µm. It was mounted on a motorized filter wheel (FRM40, OWIS GmbH, Staufen, Germany), which allows moving the filter in or out of the beam on a sub-second time scale.

To demonstrate the feasibility of the proposed KES CT technique, two CT scans of an excised porcine kidney together with a kidney stone were performed. One scan was done with the iodine filter in the beam, while the other was performed with the full spectrum of the MuCLS. At the time of the measurements, the MuCLS produced a flux of 1.3 × 10^10^. Each scan had 1000 equally-spaced projections over 360 degrees and an acquisition time of 44 ms per projection. Each scan therefore had a total acquisition time of 44 s. Together with the time needed for the movement/turning of the sample stage and for detector communication, the total scan time added up to 10 min per scan. The kidney was set into a plastic beaker glass and undiluted iodine-based contrast agent (IMERON 400 MCT, Bracco Imaging, Germany) was injected into the renal arteries. The kidney was refrigerated prior to the measurements and placed in cold water during the experiment to slow down the diffusion of the iodine-based contrast agent out of the blood vessels into the surrounding tissue. The kidney stone, made of calcium oxalate (90% whewellite and 10% weddellite), was placed into the skin surrounding the kidney (compare Fig. 5a). The data was acquired with a flat panel detector (Dexela 1512, PerkinElmer, Inc., USA) with a Gd_2_O_2_S scintillator and a pixel size of 74.8 × 74.8 μm^2^. The CT scans have an effective pixel size of 70 × 70 μm^2^. The tomographic reconstruction was performed with a statistical iterative reconstruction algorithm^46^. Only after the reconstruction, CT scans were subtracted slicewise. Thereby, the iodine filtered scan was subtracted from the unfiltered scan. To visualize the kidney stone without blood vessels, the unfiltered CT scan was subtracted from the iodine filtered scan, which was multiplied with an energy correction coefficient, calculated by the ratio of the mean energies of the scans to the power of three to correct for the energy dependence of the photoelectric effect. The kinetic energy released per unit mass in air (air kerma) was calculated to 0.16 mGy/s for the unfiltered CT scan and 0.07 mGy/s for the filtered scan and hence 10.32 mGy for the total measurement time of 88 s. Using the conversion coefficients for air kerma and effective dose for kidney tissue^47^, the total effective dose for both scans combined is 2.67 mSv.

For the measurements, the kidney from a 5-month old female pig of the race German landrace was taken. The animal was euthanized in strict accordance to standard guidelines of an animal experiment proposal approved by the Institutional Animal Care and Use Committee of the Technical University of Munich. The organs were removed and further used according to the 3R (reduce, refine, replace). The kidney stone was derived from a patient at the university hospital Klinikum rechts der Isar, following the common clinical practice with respect to their individual diagnosis and indication, and after the finalization of the histopathological analysis. Written and informed consent was obtained from the patient. The study was approved by the ethics committee of the Klinikum rechts der Isar, Technical University of Munich.

## Supplementary information

Captions for Supplementary Videos

Video to Figure 4 a)

VIdeo to Figure 4b)

Video to Figure 4c)

## Data Availability

All relevant data will be made publicly available from mediaTUM (in preparation).
