# Author Correction: K-edge Subtraction Computed Tomography with a Compact Synchrotron X-ray Source

**DOI:** 10.1038/s41598-020-61222-9

**Published:** 2020-03-04

**Authors:** Stephanie Kulpe, Martin Dierolf, Benedikt Günther, Madleen Busse, Klaus Achterhold, Bernhard Gleich, Julia Herzen, Ernst Rummeny, Franz Pfeiffer, Daniela Pfeiffer

**Affiliations:** 1 Chair of Biomedical Physics, Department of Physics, Technical University of Munich, James-Franck-Straße 1, 85748 Garching, Germany; 2 Munich School of BioEngineering, Technical University of Munich, Boltzmannstraße 11, 85748 Garching, Germany; 3 Department of Diagnostic and Interventional Radiology, School of Medicine & Klinikum rechts der Isar, Technical University of Munich, Ismaninger Straße 22, 81675 München, Germany

Correction to: *Scientific Reports* 10.1038/s41598-019-49899-z, published online 16 September 2019

The Data Availability section in this Article is incorrect.

“All relevant data will be made publicly available from mediaTUM (in preparation).”

should read:

“All relevant data is publicly available from mediaTUM (http://doi.org/10.14459/2019mp1515285).”

