# Supplementary material for: K-edge Subtraction Computed Tomography with a Compact Synchrotron X-ray Source
Source: Sci Rep. 2019 Sep 16;9:13332. doi: 10.1038/s41598-019-49899-z (PMC6746727; doi:10.1038/s41598-019-49899-z)
Supplement: Supplementary file 1 — Captions for Supplementary Videos [file 41598_2019_49899_MOESM1_ESM.docx]

**K-edge Subtraction Computed Tomography with a Compact Synchrotron X-ray Source**

Stephanie Kulpe^(1,2)^*, Martin Dierolf^(1,2)^, Benedikt Günther^(1,2)^, Madleen Busse^(1,2)^, Klaus Achterhold^(1,2)^, Bernhard Gleich^(2)^, Julia Herzen^(1,2)^, Ernst Rummeny^(3)^, Franz Pfeiffer^(1,2,3)^ and Daniela Pfeiffer^(3)^

(1) Chair of Biomedical Physics, Department of Physics, Technical University of Munich, James-Franck-Straße 1, 85748 Garching, Germany

(2) Munich School of BioEngineering, Technical University of Munich, Boltzmannstraße 11, 85748 Garching, Germany

(3) Department of Diagnostic and Interventional Radiology, Klinikum rechts der Isar, Technical University of Munich, Ismaninger Straße 22, 81675 München, Germany

**Supplementary Information**

**S1-S3:** Supplementary videos ‘S1_a_unfiltered.mpg’, ‘S2_b_KES.mpg’ and ‘S3_c_KESCa.mpg’ show the 360° view of the histogram-based segmentation of the CT data presented in Fig. 4 of the manuscript.
